# *Paracoccidioides brasiliensis* Interferes on Dendritic Cells Maturation by Inhibiting PGE_2_ Production

**DOI:** 10.1371/journal.pone.0120948

**Published:** 2015-03-20

**Authors:** Reginaldo K. Fernandes, Tatiana F. Bachiega, Daniela R. Rodrigues, Marjorie de A. Golim, Luciane A. Dias-Melicio, Helanderson de A. Balderramas, Ramon Kaneno, Ângela M. V. C. Soares

**Affiliations:** 1 Department of Microbiology and Immunology, Biosciences Institute, São Paulo State University, UNESP, Botucatu, São Paulo, Brazil; 2 Flow Cytometry Laboratory, Hemocenter, São Paulo State University, UNESP, Botucatu, São Paulo, Brazil; 3 Department of Pathology, Botucatu Medical School, São Paulo State University, UNESP, Botucatu, São Paulo, Brazil; Institut National de la Santé et de la Recherche Médicale (INSERM), FRANCE

## Abstract

Paracoccidioidomycosis (PCM) is a systemic mycosis, endemic in most Latin American countries, especially in Brazil, whose etiologic agent is the thermodimorphic fungus of the genus *Paracoccidioides*, comprising cryptic species of *Paracoccidioides brasiliensis*, S1, PS2, PS3 and *Paracoccidioides lutzii*. The mechanisms involved in the initial interaction of the fungus with cells of the innate immune response, as dendritic cells (DCs), deserve to be studied. Prostaglandins (PGs) are eicosanoids that play an important role in modulating functions of immune cells including DCs. Here we found that human immature DCs derived from the differentiation of monocytes cultured with GM-CSF and IL-4 release substantial concentrations of PGE_2_, which, however, were significantly inhibited after challenge with *P*. *brasiliensis*. *In vitro* blocking of pattern recognition receptors (PRRs) by monoclonal antibodies showed the involvement of mannose receptor (MR) in PGE_2_ inhibition by the fungus. In addition, phenotyping assays showed that after challenge with the fungus, DCs do not change their phenotype of immature cells to mature ones, as well as do not produce IL-12 p70 or adequate concentrations of TNF-α. Assays using exogenous PGE_2_ confirmed an association between PGE_2_ inhibition and failure of cells to phenotypically mature in response to *P*. *brasiliensis*. We conclude that a *P*. *brasiliensis* evasion mechanism exists associated to a dysregulation on DC maturation. These findings may provide novel information for the understanding of the complex interplay between the host and this fungus.

## Introduction

Paracoccidioidomycosis (PCM) is a systemic mycosis, endemic in most Latin American countries, especially in Brazil, whose etiologic agent is the thermodimorphic fungus of the genus *Paracoccidioides*, comprising cryptic species of *Paracoccidioides brasiliensis*, S1, PS2, PS3 and *Paracoccidioides lutzii* [1–3]. Hosts are infected through the respiratory tract by mycelium propagules found in soil that reach the alveoli where conidia convert to yeast, the infective form [4, 5]. Thereafter, yeasts can disseminate by lympho-haematogenous route, inducing a disease with a wide spectrum of symptoms in a small number of individuals suggesting that in most of exposed subjects innate and adaptive mechanisms efficiently assure resistance [6, 7]. Studies in human and animals have shown that resistance to *P*. *brasiliensis* is determined by a Th_1_ response [8–15] with TNF-α and IFN-γ playing an essential role [16], while the susceptibility involves a Th_2_ response with main participation of IL-4, IL-5, IL-10 and TGF-β [17–19]. Recently, an important study showed that individuals with PCM infection (PI) present a predominant Th_1_ response while those with chronic/adult form (AF) develop a Th_17_/Th_22_ pattern. The acute, subacute/juvenile form (JF) is the most severe form of the disease being characterized by Th_2_/Th_9_ type response [20]. Although aforementioned studies have shown that resistance/susceptibility in PCM can be explained by the involvement of different subpopulations of CD4^+^ cells, the mechanisms leading to preferential induction of any subpopulation are still unclear and those involved in the initial interaction of the fungus with cells of the innate immune response, mainly the dendritic cells (DCs), deserve to be studied.

Dendritic cells have the primary function to bind, capture, kill and process microorganisms, and migrate to peripheral lymphoid organs where they mature for efficiently triggering and instructing a T cell response [21–23]. Thus, the nature of the DCs/microorganisms interactions defines the predominant type of effector T cells. However, for the most of organisms, including fungi, the receptors signaling pathways and other molecules involved in this modulation are poorly understood. Among them pattern recognition receptors (PRRs) take an important role in the binding of microorganism to DC [24, 25] triggering events that modulate the phagocytosis, antigen processing, induction of oxidative metabolism, and cytokine production [26–29].

*P*. *brasiliensis* induces migration of DCs from murine lungs to lymph nodes, however, bone marrow-derived DCs of these mice have low capability to induce a Th_1_ response [30], confirming previous studies on susceptible animals showing that fungus inhibits the expression of class II MHC molecules as well as IL-12 and TNF- α production by DCs [31], while induces IL-10 production by regulatory DCs [32]. On the other hand, human DCs in response to *P*. *brasiliensis* express CD83, CD80, CD86, and CCR7, and produce TNF-α, IL-6 and IL-12p40 [33]. Accordingly, genes encoding the cytokines IL-12 and TNF-α and chemokines CCL22, CCL27 and CXCL10 are positively regulated in DCs infected with the fungus [34]. Calich’s group proposed that an exacerbated inflammatory early responsiveness can hinder the development of a protective specific immune response. In this context, it was observed that DCs from susceptible animals secrete high levels IL-12 and TNF-α and promote an exacerbated proinflammatory response that in turn induces T cell anergy. In opposition, in resistant animals production of proinflammatory cytokines is accompanied by high levels of TGF-β and concomitant induction of regulatory T cells. This regulated response facilitates the development of IFN-γ, IL-4 and IL-17 producing effector T cells [35]. Although these studies have elucidated some aspects of the *P*. *brasiliensis*/DCs interaction, the possible role of other potential modulators of this interaction deserves to be considered. Among these modulators we highlight the importance of eicosanoids, such as prostaglandin E2 (PGE_2_).

PGE_2_ plays an important role in modulating the immune response [36]. Its effects are preferably suppressor and can already be detected during the development of the innate immune response, since it inhibits granulocyte functions [37] as well as the phagocytosis and killing functions by alveolar macrophages [38, 39] and monocytes [40–42]. This eicosanoid by supressing NK cells activating cytokines such as IL-12 and IL-15 [43, 44] inhibits the cytolytic effector functions of these cells [45, 46]. PGE_2_ is also a potent suppressor during adaptive immune response. Its effect can be direct on T cell proliferation since it inhibits IL-2 production [47] and the expression of its receptor [48–49]. However, the most important effect of PGE_2_ on adaptive immune response is the ability regulate the balance between different CD4 responses. In this context, PGE_2_ shifts the balance from Th_1_ response toward Th_2_, by regulating IFN-γ, a Th_1_ cytokine, but not the Th_2_ cytokines IL-4 and IL-5 [50, 51]. In addition to these direct effects on CD4 T cells, the suppressive effect of PGE_2_ on Th_1_ response can also result from its action on APC cells. It inhibits IL-12 production by monocytes [52] and DCs [53, 54] as well as the expression of the receptor for this cytokine [55]. These findings were confirmed by more recent studies showing that skewed Th_2_ immune response involves expression of cyclooxygenase-2 (COX-2) in DCs [56]. Others have shown that PGE_2_ produced by DCs may be involved in regulatory T cells (T_regs_) expansion [57]. Despite all these studies demonstrating the suppressor impact of PGE_2_ during the induction of immune responses, some studies have shown the importance of this mediator in the induction of fully mature DCs capable of homing to lymph nodes and to be highly effective in priming naive T cells [58, 54, 59, 60].

According to these studies the greater or lesser ability of microorganisms to induce PGE_2_ production by DCs can influence the maturation of these cells in response to these microorganisms. Additionally, this ability can be influenced by the type of PRR to which this organism will bind in DCs. Little is known about the receptors involved in PGs production by DCs, but TLR2, dectin-1, and DC-SIGN appears to be the most involved [61].

Here, we aimed to investigate whether human DCs produce PGE_2_ in response to *P*. *brasiliensis* of high and low virulence, the role of PRRs in this production and whether these mediators modulate the DC maturation in response to the fungus.

## Material and Methods

### Subjects

Dendritic cells were differentiated from monocytes of healthy blood donors from University Hospital of the School of Medicine Botucatu, São Paulo State University, UNESP, after signature of informed consent form. The study was approved by the Institution Research Ethics Committee (registration number: 375/2011).

### Fungi

We used yeast cells of high virulent and low virulent strains of *P*. *brasiliensis* (Pb18 and Pb265, respectively). To ensure virulence, the isolate was used after three serial animal passages. Yeast cells were then maintained by weekly sub-cultivation in agar GPY medium (2% glucose, 1% peptone, 0.5% yeast extract and 2% agar medium GPY at 37°C) and used on the sixth day of culture. To obtain individual cells, the fungal suspension was homogenized with glass beads in a *Vortex* homogenizer (three cycles of 10 s), followed by sedimentation of undissolved lumps at 37°C for 5 minutes. Supernatants with most single cells were collected and counted in a *Neubauer* chamber, using a phase contrast microscope, considering bright cells as viable, since dead cells are black. Yeast cells were adjusted to 2 x 10^5^ cells/mL and only suspensions with viability ≥ 90% were used.

### Generation of monocyte-derived DCs (mo-DCs)

Peripheral blood mononuclear cells (PBMCS) were separated by Ficoll- Hypaque density gradient (Sigma-Aldrich, St. Louis, MO, USA) (centrifugation at 405 g for 30 minutes). Cells were collected and erythrocytes were eliminated by treatment with lysis buffer for 5 minutes at room temperature and 2 times washing with RPMI 1640 culture medium (Sigma-Aldrich). Then, cells were suspended in complete culture medium (RPMI 1640 supplemented with 2 mM L-glutamine, 40 mg/mL gentamicin and 10% inactivated fetal bovine serum) seeded in six-well tissue culture plates (5 x 10^6^ cells /mL) and allowed to adhere for 2 h at 37°C in an atmosphere of 5% CO_2_. Non-adherent cells were then removed by washing plates with RPMI 1640 culture medium and monocyte rich cultures were incubated with complete culture medium containing 80 ng/mL of rH IL-4 and 80 ng/mL of rH GM-CSF (R&D Systems, Inc, Minneapolis, MN, USA) for 7 days. After this period, loosely adherent cells (considered as immature dendritic cells) were collected, washed with RPMI 1640, seeded in to 24-well tissue culture plates (10^6^ cells/mL) and submitted to the different treatments. Flow cytometry assays identified the collected cells as having the CD14^low^/CD1a^high^/CD83^low^ phenotype, which is characteristic of immature DC. Cells viability was checked during the experiments by using trypan blue exclusion test.

### PGE_2_ production by DCs

Immature DCs (10^6^/mL) were challenged with Pb18 or Pb265 (2 x 10^5^ yeasts/mL) using a DCs/yeasts ratio of 5:1, or treated with LPS (5 μg/mL) for 1, 2, 4, 8, 12, 18, 24 or 48 h. Supernatants were harvested and assayed for PGE_2_ levels using a competitive enzyme-linked immunosorbent assay kit (Cayman Chemical Company, Ann Arbor, MI, USA). In some experiments, DCs were incubated for 2h with monoclonal antibodies: anti-TLR2 (2 μg/10^6^ cells), and/or anti-MR (2 μg/10^6^ cells) (monoclonal antibodies purchased from Biolegend, San Diego, CA, USA), anti-dectin-1 (3 μg/10^6^ cells) and anti-DC-SIGN (4 μg/10^6^ cells) before fungus challenge (monoclonal antibodies purchased from R&D Systems, Minneapolis, MN, USA). These concentrations were chosen because they induced the highest percentages of blockage in previous experiments (data not shown). The protocols were designed in order to block three receptors keeping only one available.

### Flow Cytometry analysis

Monocyte-derived immature DCs were phenotyped by flow cytometry for CD14 (PerCP), CD1a (FITC) and CD83 (PE) expression in a FACSCalibur^TM^ flow cytometer (BD-Becton,Dickinson and Company, San Diego, CA, USA) using the CellQuest software (BD-Becton-Dickinson, Company). In subsequent experiments immature DCs (10^6^/mL) challenged with Pb18 or Pb265 or treated with LPS and respective controls were evaluated for expression of HLA-DR (FITC), CD40 (PerCP), CD80 (FITC), CD83 (PE), CD86 (APC), CCR5 (FITC), CCR7 (PerCP) e CXCR4 (PerCP). In some experiments 100 μg/mL of exogenous PGE_2_ was added to DCs challenged with the fungus.

### Cytokine production

Immature DCs (10^6^/mL) were challenged with Pb18 or Pb265 or treated with LPS (5 μg/mL) for 48 h and culture supernatants were evaluated for IL-12p70 and TNF-α production using commercial duoset ELISA kits (BD OptEIA-Becton, Dickinson and Company). IL-12p70 production was also determined by using an other commercial duoset ELISA kit (R&D Systems) and a cytometric bead assay (CBA) (BD-Becton,Dickinson and Company). In some experiments 100 μg/mL of exogenous PGE_2_ was added to DCs challenged with the fungus.

### Statistical analysis

Statistical analysis was performed by using the *GraPhpad Prism* Version 5.01 for Windows, *GraphPad* Software, Inc. (San Diego, CA, USA). Significant differences among groups were determined by analysis of variance test (ANOVA) for dependent samples, and the averages compared by Multiple Correlations Tukey-Kramer test, assuming as true every case in which the probability of error was less than 5% (p< 0,05).

## Results

### PGE_2_ production by DCs challenged with *P*. *brasiliensis*

After confirmation that cells had a phenotype of immature DCs (CD14^low^/CD1a^high^/CD83^low^) we aimed to evaluate whether they produce PGE_2_ in response to challenge with *P*. *brasiliensis*. We tested both, Pb18 and Pb265, precisely because differences in the ability to produce PGE_2_ and the consequences of this process, for example, greater or lesser ability to induce maturation of DCs, could be one of the factors that determine the differences in virulence presented by these two strains.

**Fig 1:**
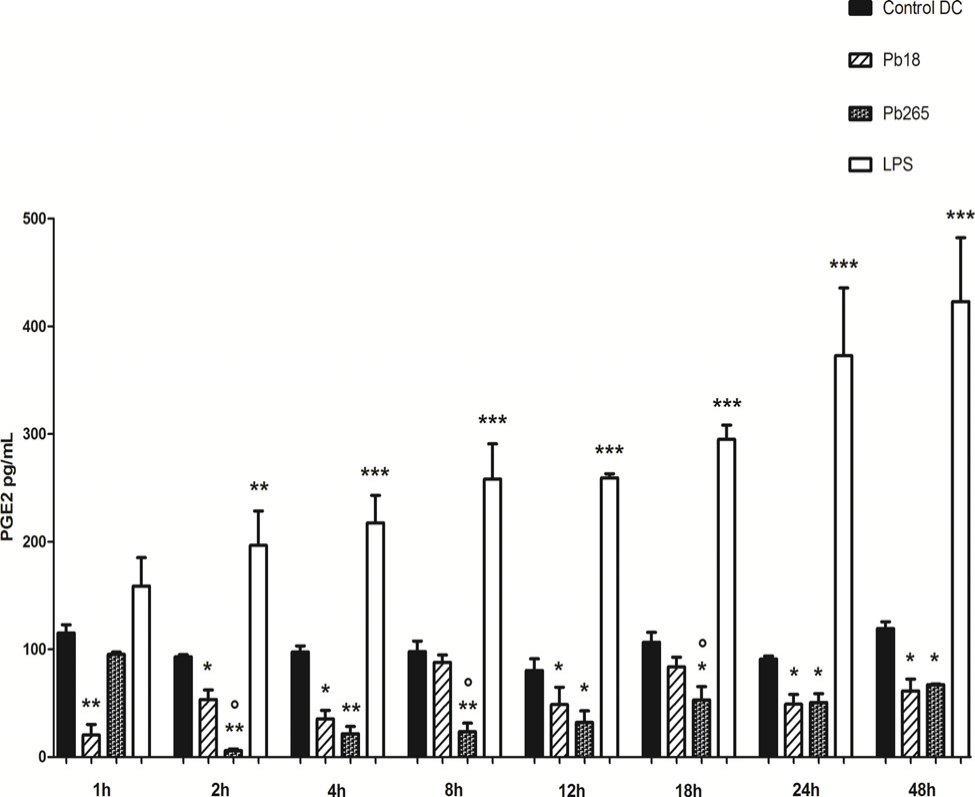
PGE_2_ production by DCs activated with LPS or challenged with Pb18 or Pb265 (DCs/Pb ratio 5:1) for different periods. The results are expressed in mean ± SD of experiments performed with cells from 5 subjects. Statistically significant differences between groups in the same period are indicated: *p< 0.05; ** p< 0.01; ***p<0.001 versus control DC; o p< 0.05 versus Pb18.

Cultures were evaluated at 1, 2, 4, 8, 12, 18, 24 and 48 h. Cell stimulation with LPS was considered as a positive control for PGE_2_ production. Despite some differences detected among the periods PGE_2_ production by control DCs was relatively stable, with minimum and maximum production of 80.304 ± 10.94 pg/mL (12 h) and 119.434 ± 6.141 pg/mL (48 h), respectively. Stimulation with LPS promoted a significant and progressive increase in PGE_2_ production (158.8 ± 26.30 pg/mL at 1 h to 372.8 ± 36.36 pg/mL at 48 h). On the other hand, challenge with Pb18 and Pb265, significantly inhibited PGE_2_ production emphasizing that in some periods (2, 8 and 18 h) inhibition induced by Pb265 was higher than Pb18 (Fig. 1).

### Involvement of MR, TLR2, Dectin-1 and DC-SIGN in PGE_2_ inhibition induced by *P*. *brasiliensis*

**Fig 2:**
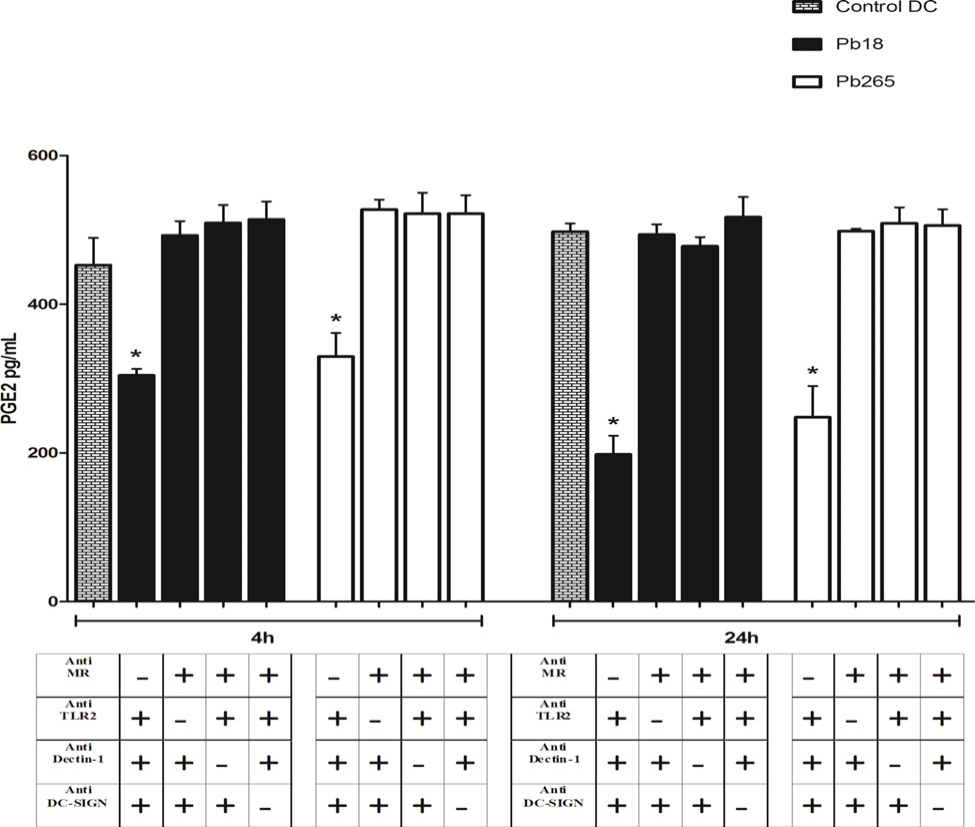
Involvement of MR, TLR2, Dectin-1 and DC-SIGN on PGE_2_ production inhibition induced by *P*. *brasiliensis* in human DCs. Cells were incubated with anti-MR, anti-TLR2, anti-Dectin-1 and/or anti-DC-SIGN monoclonal antibodies for 1 h, challenged with Pb18 or Pb265 (DCs/yeast ratio 5:1) for 4 and 24 h, and evaluated for PGE_2_ production by ELISA. The results are expressed in mean ± SD of independent experiments performed with cells obtained from 4 subjects. Statistically significant differences between groups are indicated: *p< 0.05 versus control DCs and other available receptors.

Once observed that Pb18 and Pb265 are able to inhibit PGE_2_ production by DCs, we aimed to evaluate which PRRs are involved in this process. For this purpose, we used a schedule in which three receptors were blocked by specific antibodies and only one remained available each time. After blocking, cells were challenged with Pb18 or Pb265 for 4 or 24 h. We found that PGE_2_ production was inhibited only when mannose receptor (MR) were kept available, indicating its role in the process. Individual availability of the TLR2, Dectin-1 or DC-SIGN resulted in levels quite similar or even higher than those detected for control DCs (Fig. 2).

### DCs maturation induced by *P*. *brasiliensis*

**Fig 3:**
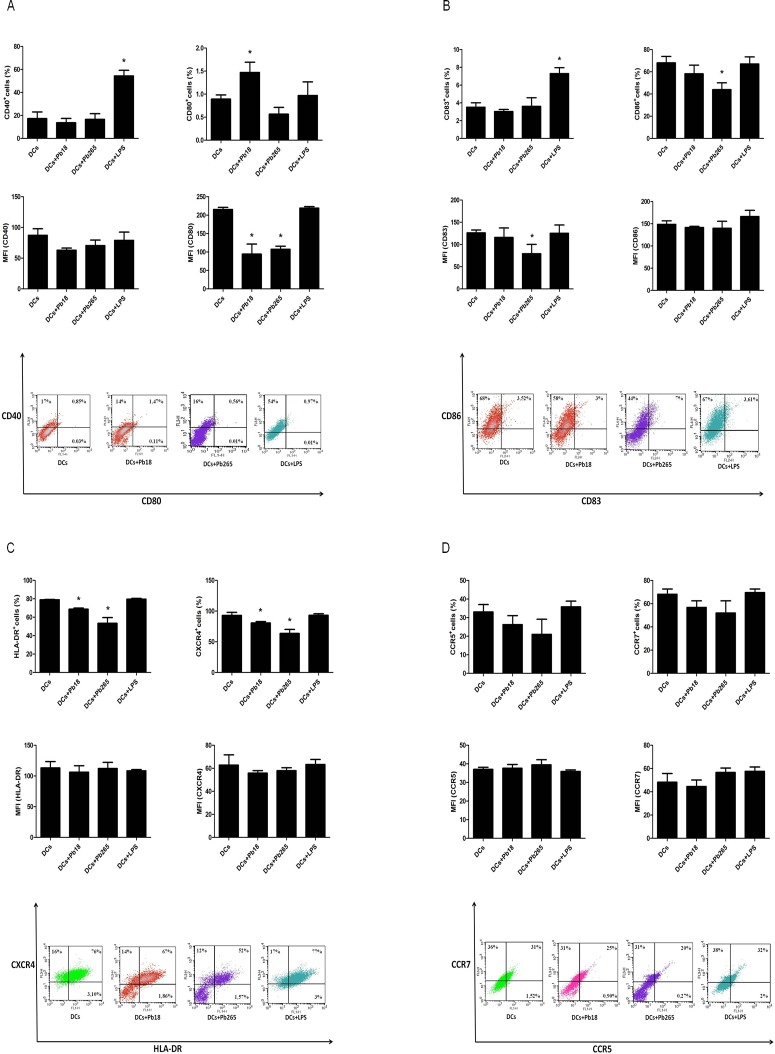
Percentage of cells, mean of fluorescence intensity (MFI), and representative dot plots relative to the expression of CD40, CD80 (A) CD83, CD86 (B), HLA-DR, CXCR4 (C) CCR5, and CCR7 (D) by DCs after challenge with Pb18 and Pb265 or activation with LPS for 48 h. The results are expressed in mean ± SD of experiments performed with cells obtained from 4 subjects. Statistically significant differences between groups are indicated: *p< 0.05 versus DCs.

Our next objective was to evaluate whether *P*. *brasiliensis* induces maturation of human DCs featured by phenotypic changes. For this purpose expression of CD40, CD80, CD83, CD86, HLA-DR, CXCR4, CCR5 and CCR7 was assessed 48 h after fungus challenge. This period was chosen as ideal for maturation analysis, because it was when we detected the higher levels of PGE_2_ after cells stimulation with LPS. We observed that challenge with Pb18 or Pb265 was not able to increase the number of DCs expressing CD40, CD83, CCR5 and CCR7. Conversely, other maturation markers CD80, HLA-DR, and CXCR4 were reduced by both strains while CD83 and CD86 were reduced by Pb265 (Fig. 3). CD80 was the only molecule with increased percentage of cells after challenge with strain Pb18. Nevertheless, when median of fluorescence intensity (MFI) was evaluated, we observed a significant decrease in response to two strains suggesting that expression of this molecule, similarly to the other above cited, was not positively regulated by *P*. *brasiliensis*. Together, these data demonstrate that the yeast fail to induce DCs maturation. The finding that the fungus does not induce increased expression of molecules involved in cells maturation or even in some cases inhibits this expression is not related to the lack of cell viability. Viability checking, frequently performed, was always above 90%. Some results with LPS are also a further demonstration of fungal effects reliability. Although expression of some molecules has not been increased probably due to problems with antibodies labeling (cytometry assays), other molecules such as CD83 and CD40 were increased in response to LPS, whereas depressed in response to the fungus.

### Cytokines production by DCs challenged with *P*. *brasiliensis*

**Fig 4:**
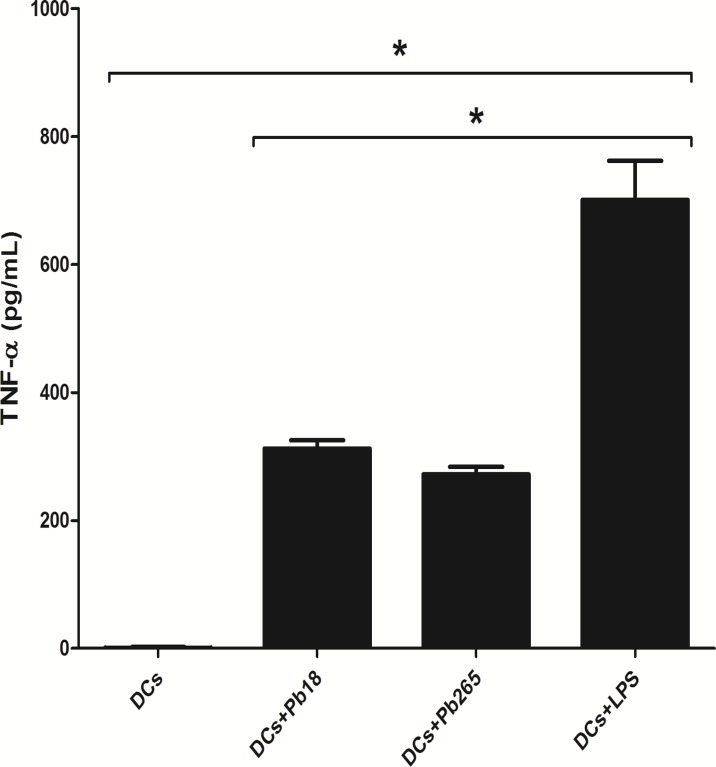
TNF-α production by DCs challenged with Pb18 or Pb265 or activated by LPS for 48 h, and measured by ELISA. The results are expressed as mean ± SD of independent experiments performed with cells obtained from 4 subjects. Statistically significant differences between groups are indicated: *p< 0.05.

In addition to the analysis of surface molecules, we aimed to evaluate IL-12 and TNF-α production, whose increases are also indicative of DCs maturation. We observed that both Pb265 and Pb18 induce DCs to release TNF- α but the levels were always lower than those induced by LPS (Fig. 4). On the contrary, we found that *P*. *brasiliensis* is not able to induce significant IL-12p70 production by DCs. Negative results were obtained by both ELISA (two different kits) and CBA assay. Overall, the results reinforce the view that the fungus is not able to induce maturation of DCs, as evidenced by the lack of changes on expression of molecules and production of some cytokines essential for activating CD4^+^ lymphocytes.

### Effect of exogenous PGE_2_ on the phenotypic maturation of DCs challenged or not with *P*. *brasiliensis*

**Fig 5:**
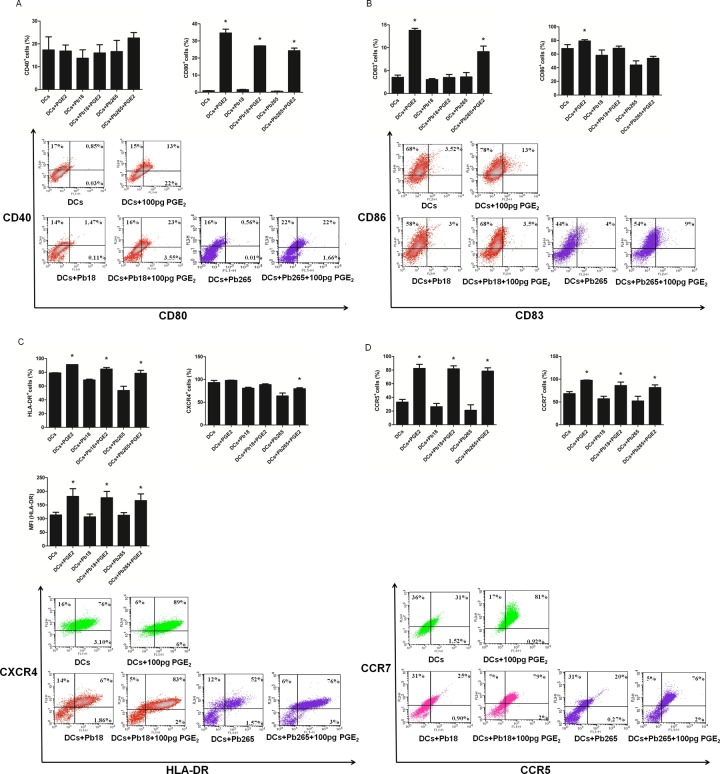
Effect of exogenous PGE_2_ on percentage of cells, mean of fluorescence intensity (MFI) relative to the expression of CD40, CD80 (A) CD83, CD86 (B), HLA-DR, CXCR4 (C) CCR5, and CCR7 (D) by DCs after challenge or not with Pb18 and Pb265. The results are expressed in mean ± SD of experiments performed with cells obtained from 4 subjects. Statistically significant differences between groups are indicated: *p< 0.05 versus without PGE_2_.

With exception of CD40 and CXCR4, exogenous PGE_2_ significantly increased the percentage of control cells (not challenged) expressing all the tested molecules (CD80, CD83, CD86, HLA-DR, CCR5, and CCR7) which confirms the effect of this mediator on positively modulating phenotypic maturation of DCs. Of most importance, it also increased the percentage of cells challenged with Pb18 and Pb265 expressing all the molecules, although the results for CD40, CD86 e CXCR4 (for Pb265) were not significant. In relation to HLA-DR the results detected for control and challenged cells were confirmed by MFI assays (Fig. 5).

**Fig 6:**
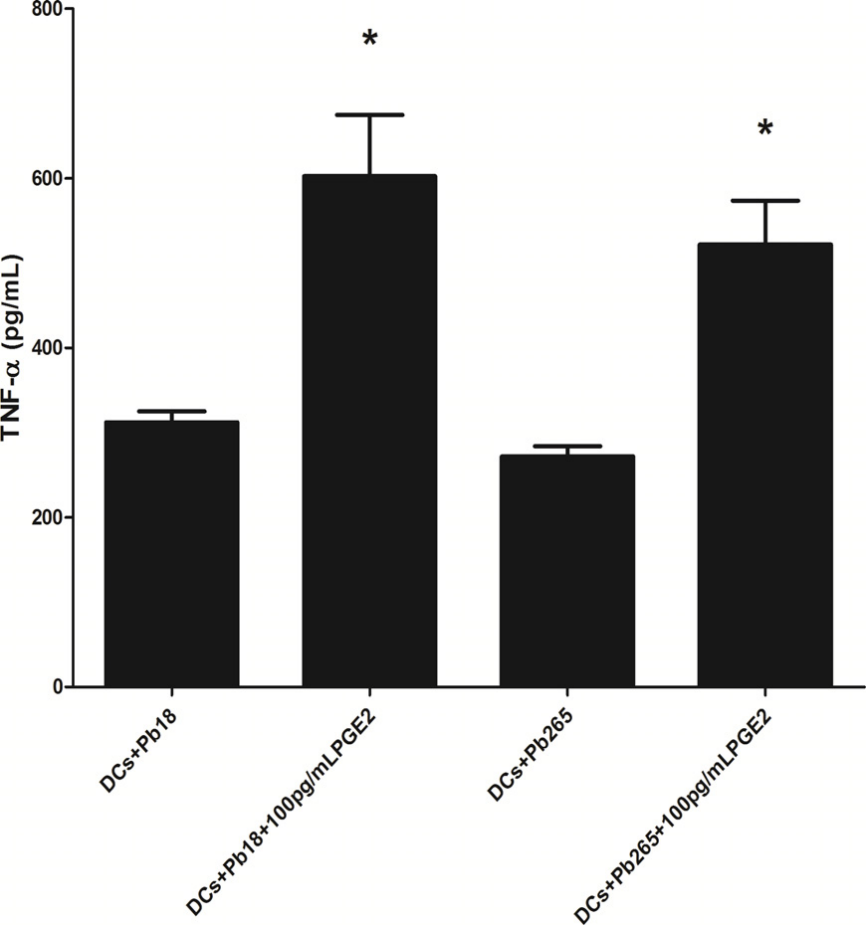
Effect of exogenous PGE_2_ on TNF-α production by DCs challenged with Pb18 or Pb265 by 48 h and evaluated by ELISA. The results are expressed in mean ± SD of experiments performed with cells obtained from 4 subjects. Statistically significant differences between groups are indicated: *p< 0.05 versus without PGE_2_.

In addition to modulation of DCs phenotype, exogenous PGE_2_ also promote alterations in TNF-α production (Fig. 6), whose *levels* after treatment were similar to those induced by LPS (Fig. 4). On other hand, DCs did not produce IL-12p70 even after exogenous PGE_2_ treatment. Taken together, data on DC phenotype and cytokine production suggest that *P*. *brasiliensis* fails to induce DC maturation, at least in part because PGE_2_ production is inhibited.

## Discussion

Studies on the modulatory role of PGE_2_ on host immune response to *P*. *brasiliensis* are scarce, particularly in relation to DCs. In this context, this study aimed to evaluate whether human DCs produce PGE_2_ in response to challenge with high and low virulent strains of *P*. *brasiliensis*, the involvement of PRRs in this process, as well as the modulatory role of this eicosanoid on maturation of these cells. These objectives were supported by previous results from our laboratory showing that Pb18 and Pb265 induce PGE_2_ production by monocytes and that this eicosanoid, in an autocrine way, inhibits antifungal activity of these cells [40–42]. Thus, this fungus would induce PGE_2_ production by monocytes as an escape mechanism from effector functions of these cells. However, contrary to detected for monocytes, in the present study we observed that both Pb18 and Pb265 inhibit PGE_2_ production by DCs. In addition, PGE_2_ inhibition was associated with impaired maturation of DCs in response to the fungus, as confirmed by low expression of CD40, CD80, CD83, CD86, HLA-DR, CXCR4, CCR5 and CCR7.

This association between inhibition of PGE_2_ and no DC maturation was strongly indicative that lack of adequate levels of this mediator is responsible for maturation failure. Experiments adding exogenous PGE_2_ to cultures challenged with the fungus confirmed this mechanism since the treatment induced an increase in the percentage of cells expressing CD80, CCR7, CCR5 and HLA-DR in response to both strains, and of CD83 and CXCR4 in response to Pb265. Our findings are corroborated by previous reports that addition of PGE_2_ to a mixture of cytokines such as IL-1β and TNF-α is essential for DCs maturation, since it considerably increases the expression of costimulatory molecules by these cells [59], and is fundamental for the expression of CCR7 [60].

CD40 is a critical coestimulatory molecule in the activation of T [62] lymphocytes, together with CD80, CD86 and mainly HLA-DR [63, 64]. CD83 molecule, besides participating in T cell activation is the main marker of DC maturation [65]. CCR7 binds the chemokines CCL19 and CCL21 derived from lymph nodes and its expression on DCs increases their ability to migrate to these organs [59, 60]. Therefore, our results showing that *P*. *brasiliensis*, by inhibiting PGE_2_ production, does not effectively induce an increase in the expression of these molecules, strongly suggest that, in vivo, the contact of fungus with DCs hinders the migration of these cells to secondary lymphoid organs, as well as their ability to activate T cells and hence initiate an adaptive immune response. Our results allow us to suggest that *P*. *brasiliensis* uses opposite mechanisms to scape monocytes and DCs responses, since increased production of PGE_2_ by monocytes inhibits their fungicidal mechanism, while impaired production by DCs avoid their maturation.

We also demonstrated that mannose receptor (MR) is the PRR involved in PGE_2_ inhibition by *P*. *brasiliensis* which emphasizes the role of this receptor in the escape mechanisms of some fungi. Accordingly, endocytosis of *Candida albicans* by MR pathway results in inhibition of NADPH oxidase pathway that is essential for fungus elimination by phagocytes [66]. Specifically in relation to *P*. *brasiliensis* previous studies suggest that it can use MR as an evasion mechanism. A particular study has shown that gp43 fraction, the immunodominant antigen of *P*. *brasiliensis*, binds to MR to inhibit phagocytic and fungicidal capacity of murine peritoneal macrophages [67]. In a recent study, we observed that *P*. *brasiliensis* phagocytized by binding to MR is able to grow inside human monocytes. In addition, IL-18 positively modulates this process by increasing fungus binding to MR receptors [68].

We also evaluated whether DCs challenged with the fungus increase their production of IL-12 and TNF-α, two important cytokines for DCs maturation. We observed that exposition of DCs to *P*. *brasiliensis* does not induce production of IL-12p70. DCs and macrophages are the main source of this cytokine in response to intracellular microorganisms [69, 70] which has a key role in the modulation of Th_1_ response. Therefore, fail of DCs to produce IL-12p70 in response to *P*. *brasiliensis* avoid their role in instructing CD4^+^ to a Th_1_ response essential for host resistance to this microorganism This result is in agreement with previous studies showing that *P*. *brasiliensis* or its main antigen gp43 inhibit IL-12 production by murine BM-DCs [71]. However, fail of DCs to produce IL-12 in response to the fungus is not associated to inhibition on PGE_2_ levels, as exogenous treatment did not result in cytokine increase. Indeed, previous studies reported that DCs activated in the presence of PGE_2_ lose their ability to secrete IL-12 [72, 67, 73].

TNF-α is a pleiotropic cytokine that regulates a broad range of biological events, including cell differentiation, proliferation, tissue development and death, as well as inflammation, innate and adaptive immune responses [74–77]. DCs maturation is highly dependent on TNF production [78–80] and we observed that fungus induce TNF-α production by DCs, but it levels were significantly increase after PGE_2_ treatment. This finding lead us to consider that inhibition of PGE_2_ results in the production of lower levels of TNF-α, that are insufficient to ensure DCs maturation.

Our findings support studies with PCM patients showing that only DCs from treated patients are effectively activated and show high expression of HLA-DR, CD86 and DC-SIGN, as well as IL-12 production. Thus, during active disease a dysregulation in DCs maturation can be detected and consequent fail to provide optimal costimulation for T cell proliferation may occur [81].

It has to be emphasized that no significant differences between responses induced by Pb18 and Pb265 were observed. The capacity of modulating host immune response by fungus strains is dependent on variations in their cell wall components [82, 16, 83] since they can account for fungus binding to different receptors of the innate immune response. More virulent strains of *P*. *brasiliensis* (as Pb18) show a smaller amount of β-glucan in their wall composition, while strains with low virulence (as Pb265) have a large amount of this carbohydrate [84] which indicates that these two strains present different capacities to bind to dectin-1, the receptor that recognizes β-glucan. In this context, as PG inhibition involves MR and not dectin-1, variations in β-glucan wall presented by two strains possibly do not interfere with this process, which can explain the similar responses detected between the two strains.

Overall, our results allow include *P*. *brasiliensis* in the growing list of microorganisms that impair DCs maturation and functions to evade a protective adaptive response. This mechanism has been detected in diseases caused by varicella-zoster [85] herpes simplex [78] vaccinia [86], measles [87], *Trypanosoma cruzi* [88], *Salmonella* [89], *Mycobacterium tuberculosis* [90, 91], *Mycobacterium leprae* [92] and *Cryptococcus gattii* [80].

In summary, we have found that *P*. *brasiliensis*, by binding to MR, inhibits PGE_2_ production by DCs which results in the production of lower TNF-α levels and consequent deregulation on DCs maturation in response to this fungus. However, the consequences of this process for delivering signals required for the induction of an efficient T cell response against the fungus, need to be determined. To answer this question, in a current study in our lab, we aimed to analysing the global transcriptional profile of DCs in response to the fungus, as well as of CD4^+^ cells in response to DCs. Together, the results will provide novel information for understanding the complex interplay between the host and *P*. *brasiliensis* and may support further therapeutic approaches.
