# Correction: *Paracoccidioides brasiliensis* Interferes on Dendritic Cells Maturation by Inhibiting PGE2 Production

**DOI:** 10.1371/journal.pone.0131380

**Published:** 2015-06-26

**Authors:** Reginaldo K. Fernandes, Tatiana F. Bachiega, Daniela R. Rodrigues, Marjorie de A. Golim, Luciane A. Dias-Melicio, Helanderson de A. Balderramas, Ramon Kaneno, Ângela M. V. C. Soares

There are errors in the Author Contributions. The correct contributions are:

Conceived and designed the experiments: RKF MAG LAD-M RK AMVCS. Performed the experiments: RKF TFB DRR HAB. Analyzed the data: RKF MAG RK. Contributed reagents/materials/analysis tools: RK AMVCS. Wrote the paper: RKF AMVCS RK. Software used in analysis: MAG.
